# Functional analysis of SARS-CoV-2 proteins in *Drosophila* identifies Orf6-induced pathogenic effects with Selinexor as an effective treatment

**DOI:** 10.1186/s13578-021-00567-8

**Published:** 2021-03-25

**Authors:** Jun-yi Zhu, Jin-Gu Lee, Joyce van de Leemput, Hangnoh Lee, Zhe Han

**Affiliations:** 1 Center for Precision Disease Modeling, Department of Medicine, University of Maryland School of Medicine, Baltimore, MD USA; 2 Division of Endocrinology, Diabetes and Nutrition, Department of Medicine, University of Maryland School of Medicine, Baltimore, MD USA

## Abstract

**Background:**

SARS-CoV-2 causes COVID-19 with a widely diverse disease profile that affects many different tissues. The mechanisms underlying its pathogenicity in host organisms remain unclear. Animal models for studying the pathogenicity of SARS-CoV-2 proteins are lacking.

**Methods:**

Using bioinformatic analysis, we found that 90% of the virus-host interactions involve human proteins conserved in *Drosophila*. Therefore, we generated a series of transgenic fly lines for individual SARS-CoV-2 genes, and used the Gal4-UAS system to express these viral genes in *Drosophila* to study their pathogenicity.

**Results:**

We found that the ubiquitous expression of Orf6, Nsp6 or Orf7a in *Drosophila* led to reduced viability and tissue defects, including reduced trachea branching as well as muscle deficits resulting in a “held-up” wing phenotype and poor climbing ability. Furthermore, muscles in these flies showed dramatically reduced mitochondria. Since Orf6 was found to interact with nucleopore proteins XPO1, we tested Selinexor, a drug that inhibits XPO1, and found that it could attenuate the Orf6-induced lethality and tissue-specific phenotypes observed in flies.

**Conclusions:**

Our study established *Drosophila* as a model for studying the function of SARS-CoV2 genes, identified Orf6 as a highly pathogenic protein in various tissues, and demonstrated the potential of Selinexor for inhibiting Orf6 toxicity using an in vivo animal model system.

**Supplementary Information:**

The online version contains supplementary material available at 10.1186/s13578-021-00567-8.

## Introduction

SARS-CoV-2 (severe acute respiratory syndrome coronavirus 2), the cause of coronavirus disease 2019 (COVID-19), is the latest in a string of outbreaks in the human population caused by highly pathogenic coronaviruses. Its high transmission rate and virulence have culminated in a raging pandemic. At the time of this writing, SARS-CoV-2 has infected nearly 80 million people globally, causing more than 1,7 million deaths (source: Johns Hopkins University). While SARS-CoV-2 shares many similarities with its predecessors, SARS-CoV (2002–2004) and MERS-CoV (Middle East respiratory syndrome-CoV; 2012), investigations have already revealed several unique features. For example, the SARS-CoV-2 spike (S) protein structure includes a distinct loop with flexible glycyl residues in place of the SARS-CoV rigid prolyl residues. Molecular modeling has indicated the SARS-CoV-2 receptor binding domain has a higher affinity for ACE2 compared to its SARS-CoV counterpart [6], which might contribute to its high virulence. These findings highlight the important roles specific viral genes can play, yet very little is known of the functions of individual SARS-CoV-2 proteins and the host systems they affect.

**Fig. 1:**
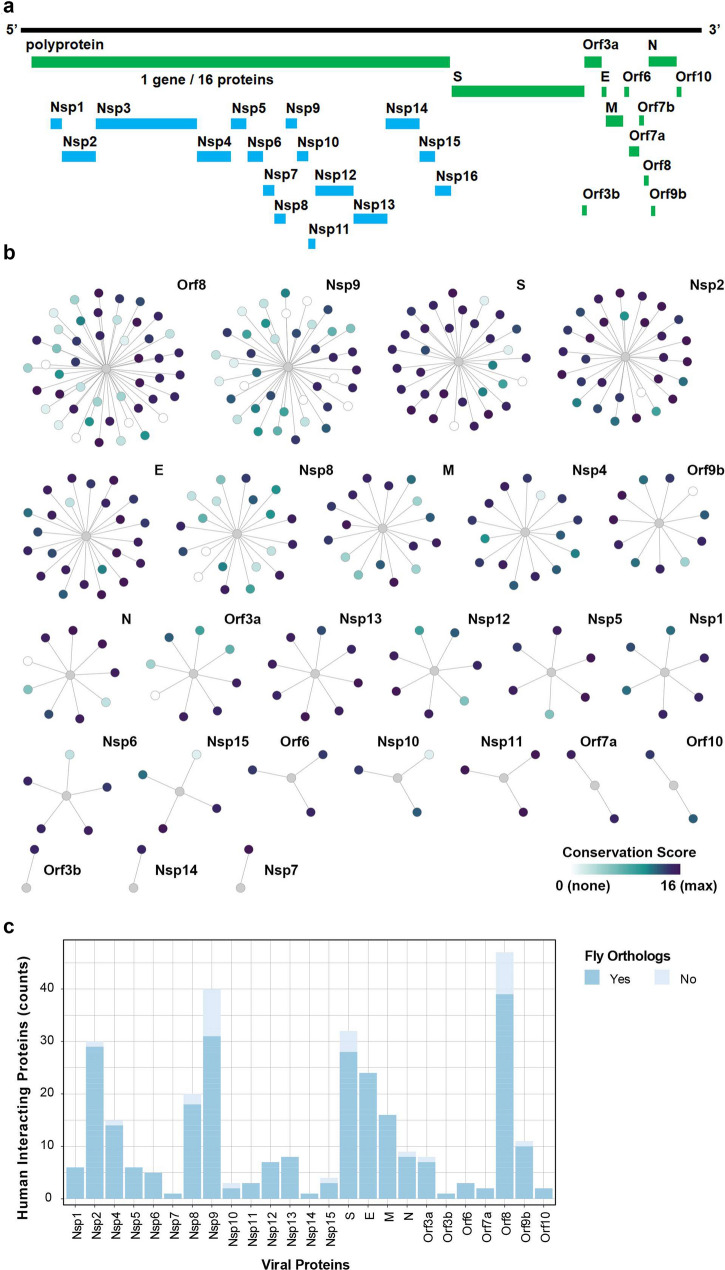
SARS-CoV-2 human protein–protein host interactome is conserved in *Drosophila*. **a** Schematic representation of SARS-CoV-2 genome. **b** Graphic display of the conservation of the SARS-CoV-2 and human host interactome in *Drosophila*. The graphs summarize the high-confidence interactions between SARS-CoV-2 proteins (bait, gray node) and human proteins (prey) described in Gordon et al. [10]. Human interacting proteins are colored based on their conservation score (i.e. DIOPT score [13]) for the best matching fly ortholog. **c** Bargraph summarizes the ortholog information displayed in (**b**). SARS-CoV-2 interacting human proteins that have fly orthologs defined or predicted by more than one source (i.e. DIOPT score >  = 2) have been included

The SARS-CoV-2 genome encodes 28 confirmed proteins (Fig. 1a). These include the polyproteins Orf1ab and Orf1a that are further cleaved into 16 non-structural proteins (Nsp1-16) that form the viral transcription/replication complex, as well as the 4 structural proteins [spike (S), envelope (E), membrane (M) and nucleocapsid (N) proteins] and 8 accessory proteins (Orf3a, Orf3b, Orf6, Orf7b, Orf8, Orf9b and Orf 10) [10, 38]. The functional significance of these accessory proteins remains largely unresolved, since they lack well-defined domain structures. These are also the least conserved between the SARS-CoV-2 and SARS-CoV viruses [10]. In fact, specific host interaction networks have been shown for each of the SARS-CoV-2 proteins [10]. In order to identify targets and develop effective therapeutics for COVID-19, it will be vital to understand how SARS-CoV-2 hijacks host pathways and which of its proteins are key for these interactions and ultimately are the primary determinants of pathogenicity.

Human models are limited to in vitro studies which lack a whole body context, while conventional animal models are not conducive to screening multiple individual proteins and many, including mice, have been hampered by lacking natural susceptibility to SARS-CoV-2 infection [34]. Therefore, we sought to establish a new animal model, namely the fruit fly (*Drosophila*). Notably, we found that the vast majority of the human host protein interactome identified for SARS-CoV-2 proteins have fly orthologs, and their related biological processes are conserved in flies (Fig. 1b and c,Additional file 1: Table S1). *Drosophila*’s ease-of-use, while offering complexity with all major organs present (including heart, lung, kidney, muscle, blood and brain), encoded by a compact genome with little redundancy, combined with the abundance of available genetic resources, has already proven effective in investigations of  several human relevant viruses in recent years [14], including HIV-1 and Zika. For example, the HIV-1 *Nef* gene was examined in *Drosophila* using the *UAS-Gal4* system [21]. The study discovered the interaction between the HIV-1 Nef protein and JNK and NF-kappaB signaling at the plasma membrane of fly wing disc cells, both are evolutionary highly conserved signaling pathways. The HIV-1 Tat protein was similarly studied in flies and found to disrupt microtubule polymerization and kinetochore dynamics via a direct interaction with tubulin [3]. This finding led to the discovery of interaction between Tat and human tubulin proteins and the important role of Tat in inducing apoptosis of human cells through targeting the microtubule network in human 293 T cells [4]. *Drosophila* studies, using the *UAS-Gal4* system to express Zika virus (ZIKV) proteins in specific tissues, have also provided significant contributions in the study of ZIKV infection and related microcephaly. One such study found that neurons employ NF-kappaB-dependent inflammatory signaling in response to ZIKV infection [26]. This, in turn, induces expression of *Drosophila* stimulator of interferon genes (dSTING) specifically in the brain of ZIKV infected flies. Activation of dSTING leads to antiviral autophagy as an innate defense to control the viral infection thereby restricting ZIKV to the brain. Another high-profile study used fly models to demonstrate that genetic variants in ANKLE2, a protein linked to hereditary microcephaly, and ZIKV protein NS4A which interacts with and inhibits Ankle2 protein function, converge on the same pathological pathways [25]. They elegantly demonstrate that Ankle2 interaction with Ballchem/VRK1 regulates asymmetric protein localization during neuroblast division, and that disruption of this pathway leads to microcephaly in both human patients due to genetic causes and flies induced by ZIKV infection. Further, a study into host transcriptomics changes following ZIKV infection in *Drosophila* adult flies, revealed the importance of the JAK/STAT signaling pathway in viral pathogenesis (Harsh, Fu, Kenney, Han, & Eleftherianos, 2020). Interestingly, further exploration of the interaction between JAK/STAT signaling and the individual ZIKV non-structural proteins revealed tissue-specific regulation of viral infection via highly conserved host signaling pathways. Together, these studies demonstrate that flies provide a powerful model to identify the prime mechanistic tissue-specific pathways used by specific viral pathogenic proteins, and that these pathways are conserved from fly to human. However, as of yet, no literature reports the use of *Drosophila* to study SARS-CoV-2.

We used *Drosophila* to identify the foremost pathogenic SARS-CoV-2 genes, and then examined their effects on host function in a whole organism. Our data revealed SARS-CoV-2 Orf6, Orf7a and Nsp6 proteins display pathogenicity in vivo. Each of these proteins individually can cause developmental lethality, reduced lifespan, defect in trachea morphology (reduced branching in fly equivalent of lung), aberrant muscle function without significant morphological changes, and reduced mitochondria in muscles. Interestingly, while all three proteins induced similar phenotypic defects, their underlying pathomechanism appeared to be different. Selinexor, an FDA-approved selective inhibitor of nuclear export and predicted to disrupt SARS-CoV-2 Orf6 interaction with the host nuclear pore system, was able to counteract virus protein induced developmental lethality in Orf6 transgenic flies, but not in flies with Orf7a or Nsp6 overexpression. Indeed, Selinexor attenuated each of the observed phenotypes in SARS-CoV-2 Orf6 transgenic flies. Taken together, these data demonstrate *Drosophila* provides a valuable resource for studying SARS-CoV-2 mechanism-of-action and pharmacological interventions. Furthermore, these findings make a strong case for the importance of studying individual SARS-CoV-2 proteins and illustrate their potential as therapeutic targets to counteract the pathogenic mechanisms culminating in COVID-19 symptomatology, as an independent and complementary therapeutic approach to currently focused treatment such as vaccine, virus entry inhibitors or virus replication inhibitors.

## Results

### SARS-CoV-2 Orf6, Nsp6, and Orf7a transgene expression causes developmental lethality and reduced longevity in flies

The SARS-CoV-2 genome encodes 28 confirmed proteins (Orf9c genetic code does not lie within the verified SARS-CoV-2 open reading frame) (Fig. 1a). Studies of the SARS-CoV-2 and human host interactome have identified high-confidence interactions between 25 SARS-CoV-2 proteins and their interacting human proteins (Fig. 1b) [10]. We examined all human proteins identified with high-confidence interactions with SARS-CoV-2 proteins and found that 90.13% have conserved homologs in *Drosophila*. Most of these conserved host proteins have predicted *Drosophila* homologs from more than one source (i.e. DIOPT score >  = 2) (Fig. 1c), suggesting that SARS-CoV-2 proteins could cause similar pathogenic effects in *Drosophila* and human cells by interacting with conserved host proteins.

To date, few studies have looked at individual viral proteins. Those that did have been limited to in vitro models. We are the first to use *Drosophila* as a whole-organism model system to carry out functional study of individual SARS-CoV-2 proteins. Using available topology and subcellular localization information from UniProt Knowledgebase [36], we identified 12 viral proteins that are most likely to instigate pathogenic host interactions. Viral proteins with main purported functions in viral entry, replication or packaging were not included, but those with unknown functions are prioritized. Twelve SARS-CoV-2 proteins (Nsp1, Nsp2, Nsp3, Nsp6, Orf3a, Orf3b, Orf6, Orf7a, Orf7b, Orf8, Orf9b and Orf10) were thus selected for the first round of functional analysis in *Drosophila*. We generated UAS-based transgenic fly lines carrying each of these 12 SARS-CoV-2 genes. The ubiquitous enhancer Tubulin (Tub) is active in all tissues and throughout development from embryonic stages, therefore we used the Tub-Gal4 to drive ubiquitous expression of individual SARS-CoV-2 gene to study their functions and pathogenicity.

**Fig. 2:**
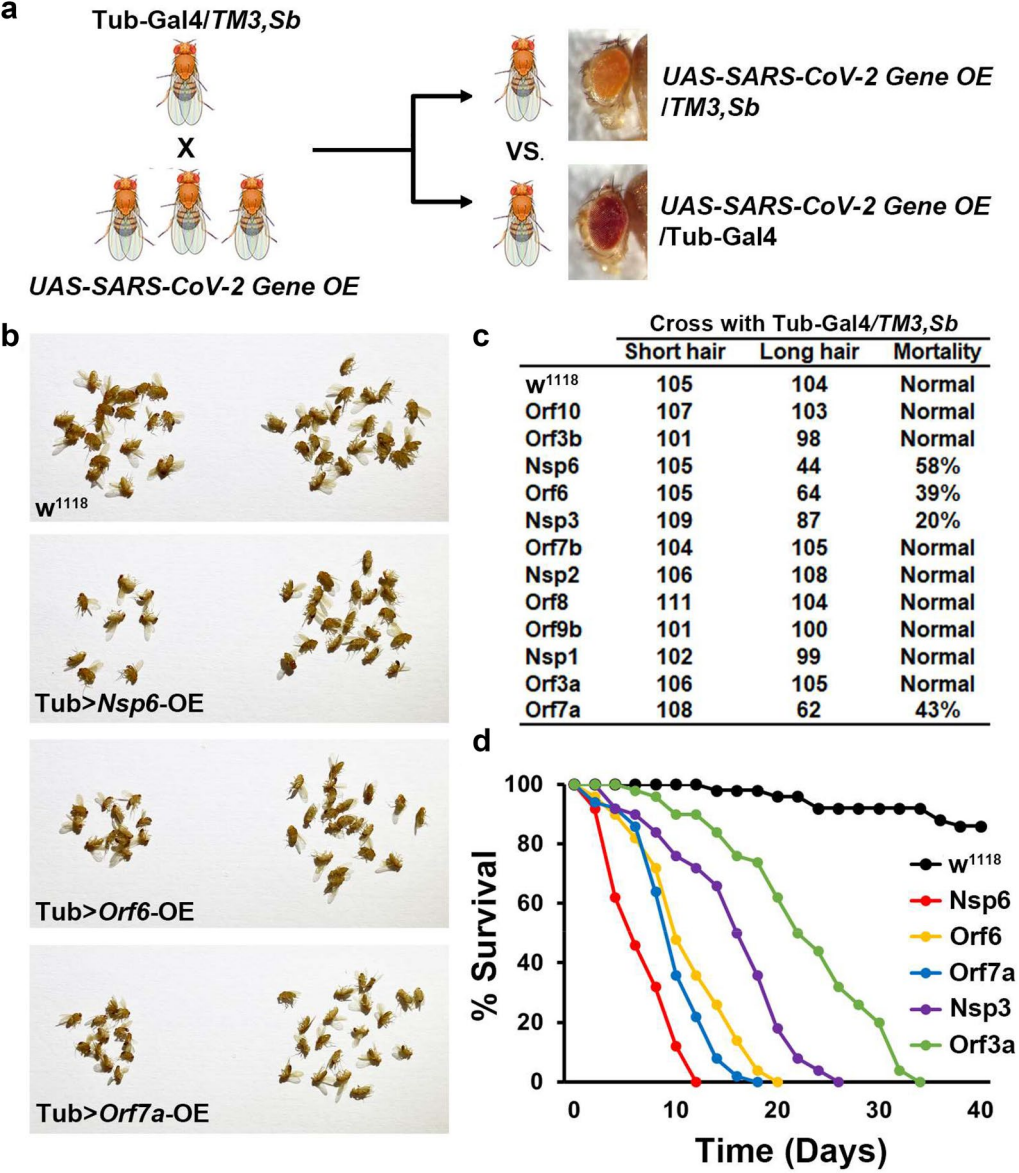
SARS-CoV-2 Nsp6, Orf6 and Orf7a transgene expression causes developmental lethality. **a** Schematic representation of genetic screen to identify individual SARS-CoV-2 genes with pathogenic effect. **b** Images of adult progeny emerging from pupa stage from cross in **a**, distinguished by carrying the balancer (*TM3, Sb*; orange eyes and short hair on back; no viral transgene expression) or with expression of the SARS-CoV-2 gene driven by the ubiquitous Tubulin (Tub) enhancer (red eyes and long hair). *w*^1118^ is a wild type control. **c** Quantification of mortality rate prior to eclosion for the individually expressed SARS-CoV-2 genes from the cross in **a**. Mortality calculated as: (long hair − short hair) / short hair × 100. **d** Graph displaying lifespan data for adult flies carrying SARS-CoV-2 Nsp6, Orf6, Orf7a, Nsp3 or Orf3a transgenes. *w*^1118^ is a wild type control. N = 100 flies per group. Abbreviations: E, envelope protein; M, membrane protein; N, nucleocapsid protein; Nsp, non-structural protein; OE, overexpression; Orf, accessory protein; S, spike protein; Sb, stubble TM3, chromosome 3

For this assay, male and female flies of the designated genotypes were crossed to produce progeny carrying the *UAS-SARS-CoV-2 gene* construct, with ubiquitous expression of the individual virus gene driven by *Tub-Gal4* (red eyes, long hair), or the balancer (orange eyes, short hair) (Fig. 2a). Ratios of these two sibling genotypes among the progeny would be 1:1 if the ubiquitous expression of a viral gene does not cause any viability defect, and less than 1 if it does. We found high mortality among flies expressing SARS-CoV-2 Orf6, Nsp6 or Orf7a, and a moderate increase in mortality among Nsp3 transgenic flies (Fig. 2b and c). In addition, we monitored fly lifespan. Under typical maintenance conditions, the majority of wild type flies survive 50–60 days. Orf6 and Orf7a expression flies displayed reduced lifespan such that all flies had died by 20 and 18 days, respectively (Fig. 2d). Nsp6 expression was associated with a severe lifespan reduction such that flies survived 12 days at most (Fig. 2d). Nsp3 which induced more moderate developmental lethality, also considerably reduced fly lifespan to 26 days. Interestingly, though Orf3a expression did not cause developmental lethality in our mortality index, it did shorten adult fly lifespan (34 days maximum), which suggests it may induce more moderate levels of toxicity or affect adult fly pathways. Together, these results indicate that Orf6, Nsp6 and Orf7a might be primary determinants of SARS-CoV-2 pathogenicity.

### SARS-CoV-2 Orf6, Nsp6 and Orf7a transgene expression leads to reduced branching of *Drosophila* trachea

**Fig. 3:**
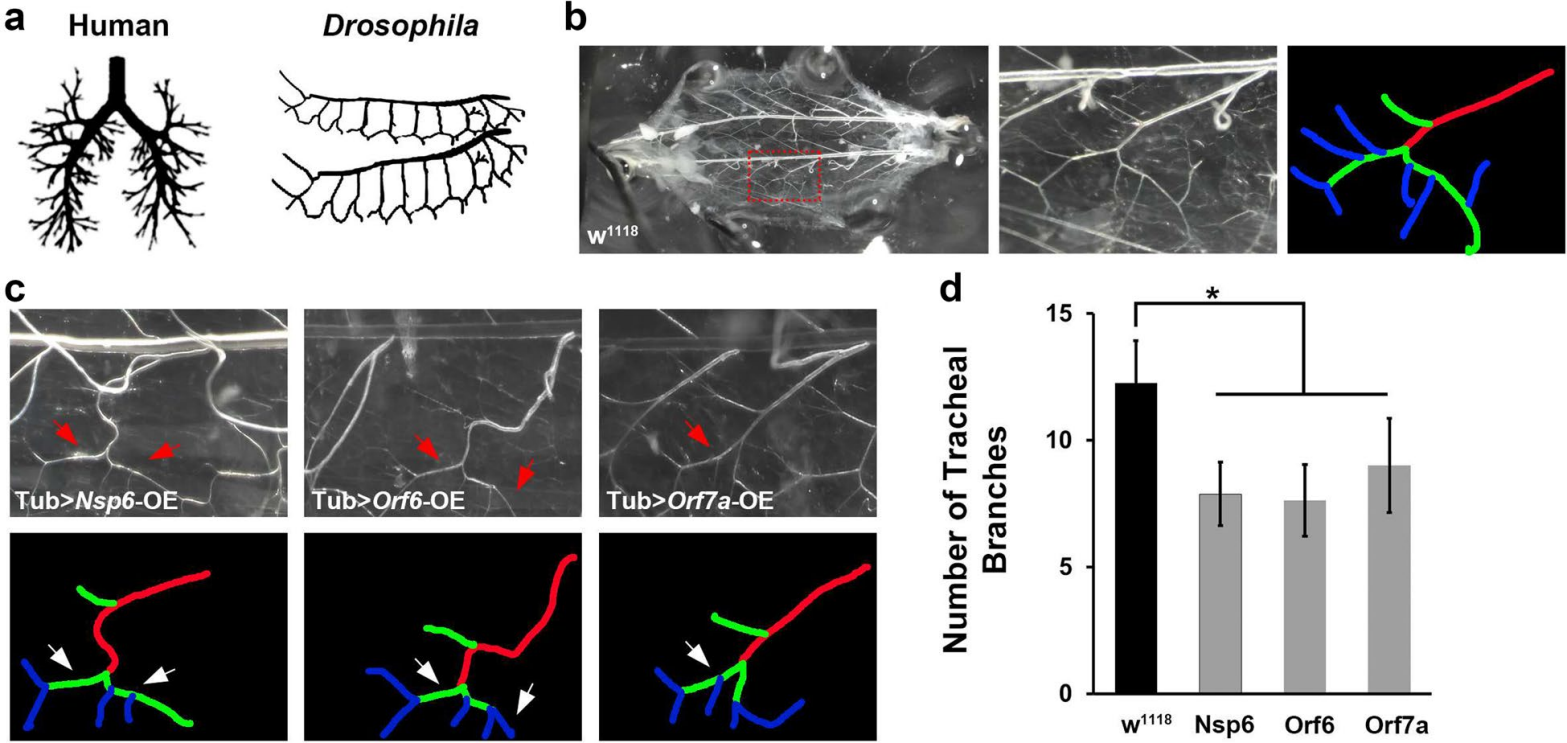
SARS-CoV2 Nsp6, Orf6 and Orf7a transgene expression leads to reduced tracheal branching in *Drosophila*. **a** Graphic representation of branching in human lung (left) and *Drosophila* trachea (right). **b** Typical tracheal branches in wild type larva (*w*^1118^). Left panel, overview of complete *Drosophila* trachea system. Red box denotes area shown at higher magnification in middle panel, as well as area covered in images shown in **c**. Right panel, labeling of tracheal branches: red, central branch; green, class I terminal branch; and, blue, class II terminal branch. **c** Representative images of tracheal branches (taken at location equivalent to red box in **b** in SARS-CoV-2 Nsp6, Orf6 or Orf7a overexpression (OE) transgenic flies. Arrow indicates a missing class II terminal branch based on wild type (*w*^1118^ shown in **b**). Tracheal branches are labeled to indicate the central branch (red), class I terminal branch (green), and class II terminal branch (blue). **d** Quantification of tracheal branch numbers in one segment. N = 6 larvae per group. Results are presented as mean ± SD. Statistical significance (*) is defined as P < 0.05

COVID-19 is an acute respiratory disease which is characterized by pneumonia when disease progresses, as well as additional complications. Due to its primary effect on the lungs, we tested whether expression of SARS-CoV-2 Orf6, Nsp6 and Orf7a (the three most pathogenic viral proteins based on our *Drosophila* viability assays), would lead to changes in the *Drosophila* tracheal system, which is structurally and functionally similar to the human lung and shares remarkable resemblance in branching morphology compared to the human lung morphology (Fig. 3a). In wild type *Drosophila* larvae, the tracheal system contains a central branch and multiple classes of terminal branches (Fig. 3b). SARS-CoV-2 Nsp6, Orf6 and Orf7a expression led to dramatically reduced numbers of class II terminal branches, while the central branch and class I terminal branches appeared unchanged (Fig. 3c and d). The changed morphology could result in a functional defect of the fly tracheal system and possibly contribute to the reduced developmental viability we observed in these flies.

### SARS-CoV-2 Nsp6, Orf6 and Orf7a transgene expression results in dysfunctional muscle and reduced presence of mitochondria

**Fig. 4:**
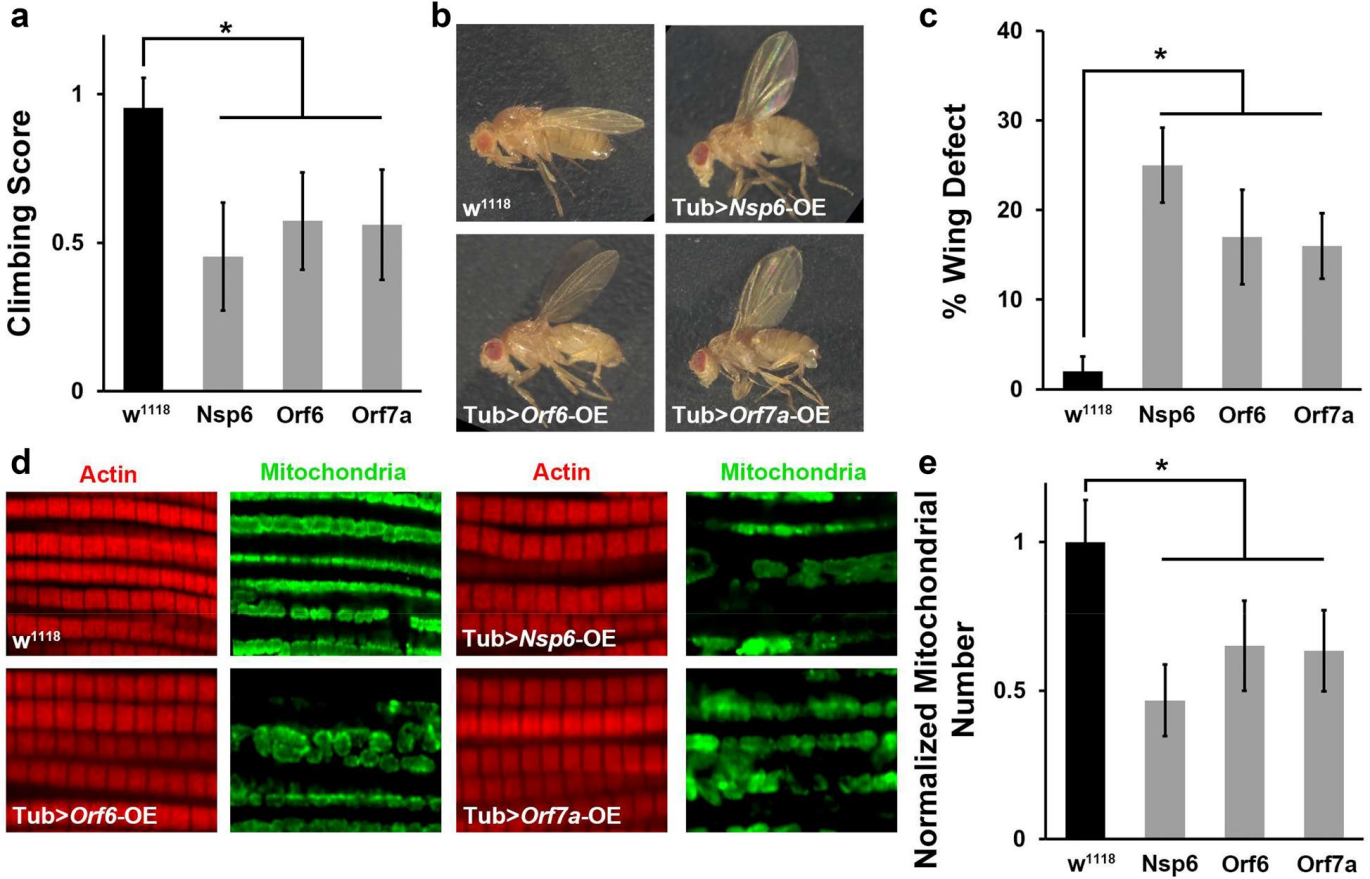
SARS-CoV-2 Nsp6, Orf6 and Orf7a transgene expression in *Drosophila* results in locomotion defect and reduced mitochondria. **a** Quantification of climbing ability in SARS-CoV-2 Nsp6, Orf6 and Orf7a transgenic flies. N = 30 flies per group. **b** Representative images of typical (wild type, *w*^1118^) and “held-up” wing phenotype in SARS-CoV-2 Nsp6, Orf6 and Orf7a expression flies. **c** Quantification of flies with “held-up” wing phenotype (i.e. % Wing Defect). Four replicates, each replicate N = 50 flies per group. **d** Representative images of indirect flight muscle (labeled with Phalloidin, red) and mitochondria (labeled with ATP5A, green) morphology in SARS-CoV-2 Nsp6, Orf6 and Orf7a flies. **e** Quantification of the number of mitochondria in the fly indirect flight muscle of both wings, normalized based on wild type (*w*^1118^) counts. N = 10 flies per group. The results are presented as mean ± SD. Statistical significance (*) is defined as P < 0.05

We examined whether Nsp6, Orf6 and Orf7a expression affected fly locomotion ability. A climbing assay revealed locomotion defects in SARS-CoV-2 Nsp6, Orf6 and Orf7a transgenic flies (Fig. 4a). Interestingly, we observed flies with an “held-up” wing position phenotype among those expressing SARS-CoV-2 Nsp6, Orf6 or Orf7a (Fig. 4b and c). The “held-up” wing defect is typically due to a defect of the indirect flight muscle. Hence, we took a closer look at indirect flight muscle morphology in the Nsp6, Orf6 and Orf7a transgenic flies, but did not observe any significant morphological changes in muscle fiber (Fig. 4d). However, when studying the mitochondria in the indirect flight muscle of these flies, we found their numbers were dramatically reduced (Fig. 4d and e). Mitochondria are essential for muscle function and their significant reduction could explain both the “held-up” wing phenotype and the reduced climbing ability of the adult flies. These data suggested SARS-CoV-2 Nsp6, Orf6 and Orf7a may affect muscle function by reducing the available function mitochondria. Additional studies are needed to understand the full mechanism underlying these findings and their implications.

### Selinexor attenuates SARS-CoV-2 Orf6 induced developmental lethality, aberrant tracheal branching, locomotion defect and reduced mitochondria

**Fig. 5:**
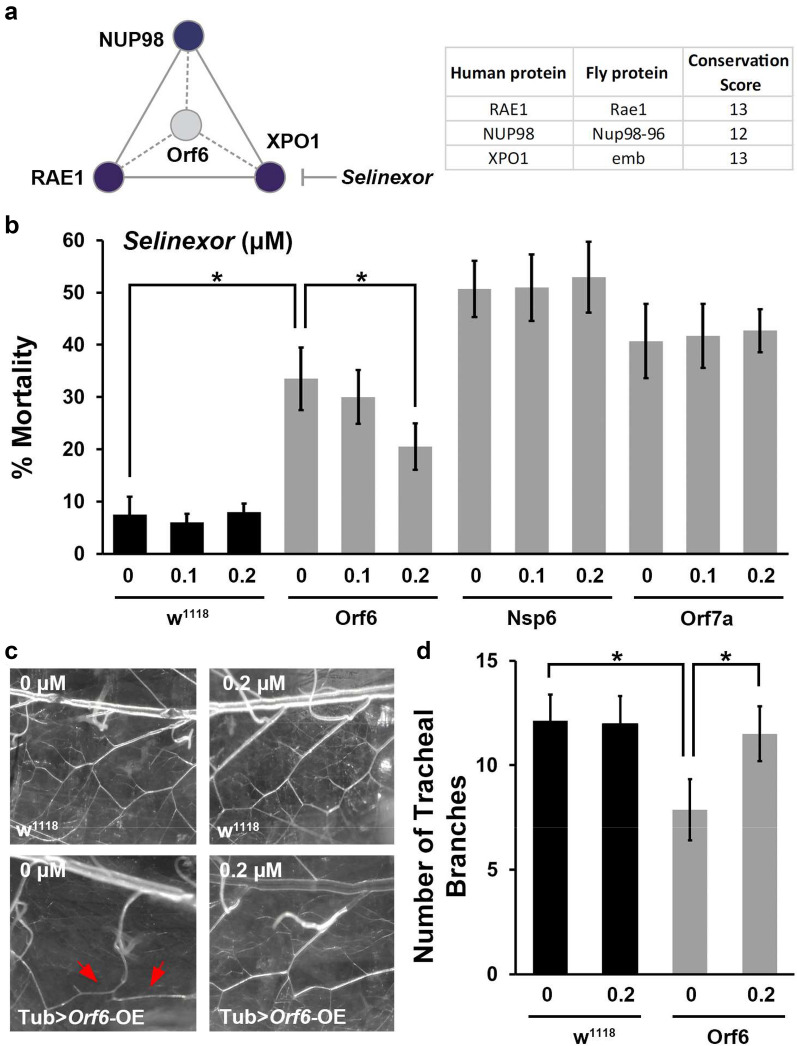
Selinexor attenuates fly developmental lethality and reduction in tracheal branching induced by SARS-CoV-2 Orf6 transgene expression. **a** Graphic presentation of conservation SARS-CoV-2 Orf6 interaction with human host proteins. Graph summarizes the high-confidence interactions between SARS-CoV-2 Orf6 protein and human proteins described in Gordon et al. [10] and our study [22]. Human interacting proteins are colored based on their conservation score (i.e. DIOPT score [13]) of the best matching fly ortholog. Numeric conservation scores human-fly proteins have been provided in the table. **b** Quantification of mortality rate prior to eclosion for wild type (*w*^1118^) or SARS-CoV-2 Orf6, Nsp6 or Orf7a transgenic flies [overexpression (OE) driven by ubiquitous enhancer Tubulin (Tub)] following treatment with different doses of Selinexor. Mortality calculated as: (long hair − short hair) / short hair × 100. Four replicates, each replicate N = 50 flies per group. **c** Representative images of tracheal branches (taken at location equivalent to red box in Fig. 3b) in wild type (*w*^1118^) and SARS-CoV-2 Orf6 overexpression (OE) transgenic flies, with (0.2 μM) or without (0 μM) Selinexor treatment. Arrow indicates a missing class II terminal branch based on typical wild type pattern. **d** Quantification of tracheal branch numbers in one segment for observations in (**c**). N = 6 larvae per group. Results are presented as mean ± SD. Statistical significance (*) is defined as P < 0.05

**Fig. 6:**
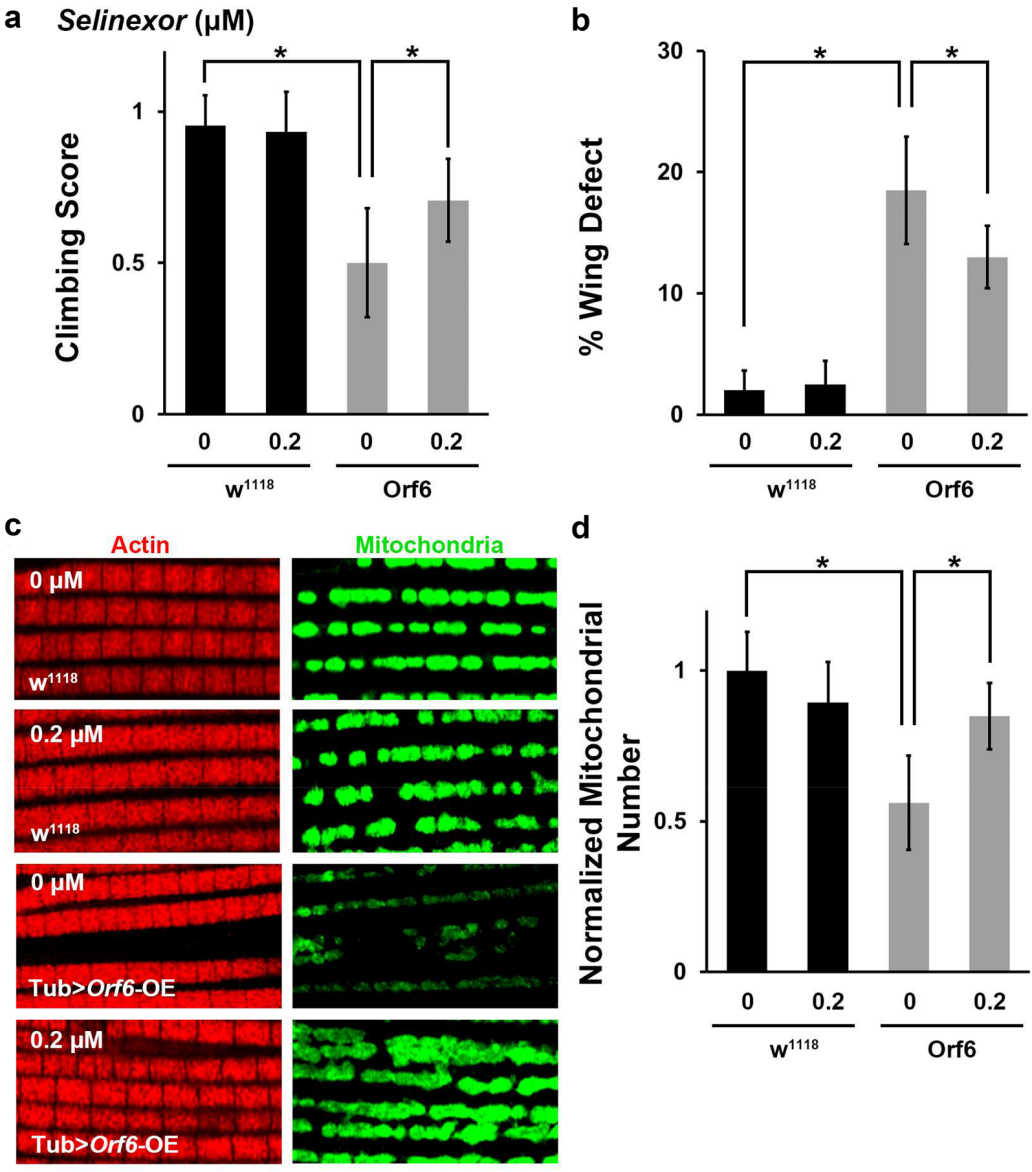
Selinexor attenuates locomotion defect and reduced mitochondria caused by SARS-CoV-2 Orf6 transgene expression in flies. Comparative assays of phenotypes in wild type (*w*^1118^) and SARS-CoV-2 Orf6 transgenic [overexpression (OE) driven by ubiquitous enhancer Tubulin (Tub)] flies following treatment with Selinexor (0.2 μM). **a** Quantification of climbing ability. N = 30 flies per group. **b** Quantification of flies with “held-up” wing phenotype (i.e. % Wing Defect). Four replicates, each replicate N = 50 flies per group. **c** Representative images of indirect flight muscle (labeled with Phalloidin, red) and mitochondria (labeled with ATP5A, green) morphology. **d** Quantification of the number of mitochondria in the fly indirect flight muscle, normalized based on wild type (*w*^1118^) counts. N = 10 flies per group. Results are presented as mean ± SD. Statistical significance (*) is defined as P < 0.05

Orf6 was found to interact with the nuclear export complex (RAE1 and NUP98) [10]. Our proteinomics analysis using Orf6-expressing human cells identified that Orf6 interacts with XPO1, a key component of the nuclear export complex [22]. Selinexor is an FDA-approved inhibitor of nuclear export [16]. Selinexor functions through direct binding to XPO1 and inhibit its activity [33]. These Orf6-binding host proteins (RAE1, NUP98 & XPO1) are highly conserved from flies to humans (Fig. 5a). Therefore, we tested whether Selinexor could rescue the various phenotypes observed in SARS-CoV-2 Orf6 overexpression flies. The flies were treated with different Selinexor doses (0, 0.1, 0.2 and 0.5 μM) from larval stage. We found 0.2 μM, but not 0.1 μM, Selinexor treatment attenuated the developmental lethality caused by Orf6, but not the lethality observed in Nsp6 or Orf7a transgenic flies (Fig. 5b). At 0.5 μM, the highest dose tested, Selinexor was toxic to the flies and caused complete lethality in the Orf6 transgenic and wild type (*w*^1118^) flies (data not shown). Further, show 0.2 μM Selinexor treatment in the flies significantly attenuated SARS-CoV-2 Orf6 induced reduction in tracheal branching (Fig. 5c and d), as well as the locomotion defect, “held-up” wing phenotype and mitochondrial loss observed in the indirect flight muscle (Fig. 6a–d). Together these findings indicate that Selinexor, and possibly other nuclear transport inhibitors, might provide an effective targeted treatment strategy for SARS-CoV-2 Orf6 protein induced cellular damage through blocking viral interaction with the host nuclear pore complex.

## Discussion

SARS-CoV-2, like most viruses, relies heavily on host functions to complete its life cycle. It encodes structural proteins (S, E, M and N) that provide a capsule structure for the virion, non-structural proteins (Nsp1-16) that interact with and modify host protein systems for virus transcription and replication, and accessory proteins (Orfs). The latter appear more variable across viruses, even within the same genus, and their functions are less understood; some are required for replication, while others are engaged in transforming the host environment to be more conductive of virus replication and spread. For example, by evading or counteracting the host immune response. Given their highly specialized functions and host interactions, it seems reasonable to assume some viral proteins are more pathogenic than others. Indeed, this has been shown for HIV-1 [8], ZIKV [37], SARS-CoV [17, 19, 20, 30, 40] and for SARS-CoV-2 [22]. Toxicity in an in vitro system does not capture the complexity of pathogenicity in a whole organism, nor does it provide any indication as to what tissues might be most infected. Animal studies for SARS-CoV-2 have been limited [2, 7, 12, 15, 29, 39], and few if any have looked at individual proteins. The *Drosophila* system is well-established and has been instrumental in identifying some of the primary detrimental virus proteins and their interaction with host pathways in HIV-1 and ZIKV infections [3, 11, 14, 21, 25, 26].

Encouraged by the considerable conservation of the SARS-CoV-2 virus-host interaction network proteins between human and fly (Fig. 1), we tested toxicity of individual viral proteins in *Drosophila*. We generated transgenic fly lines expressing each of the SARS-CoV-2 genes predicted to be most likely pathogenic based on available resources. SARS-CoV-2 Orf6, Nsp6 and Orf7a proofed most pathogenic based on mortality at eclosion (*i.e.* prior to the adult fly emerges) and longevity (Fig. 2), these were also most toxic in our human in vitro screen using HEK 293 T cells [22]. Nsp3 expression in fly also caused developmental lethality and reduced longevity, albeit with smaller effect than the main pathogenic proteins. Notably, Nsp3 was not found to affect viability of the human cells [22]. This does not exclude possible toxicity to cellular pathways that do not directly affect apoptosis and reflects the added value of looking in a live animal model which captures effects on all cell types and tissues. Orf3a, on the other hand, displayed no detectable change in developmental mortality, however it significantly reduced fly lifespan and showed cytotoxicity in HEK 293 T [22]. These data suggest that in fly, Orf3a either induced relatively moderate toxicity or interacts with, and affects, host systems that play a role after adult hatching. Taken together, these findings show the fly system can accurately capture SARS-CoV-2 individual protein pathogenicity.

Similar to host tissue-specific effects based on tissue differences in proteins and pathways, the individual virus proteins are equally important in determining the effects on a tissue. Our data revealed similar phenotypic read-outs for SARS-CoV-2 Orf6, Nsp6 and Orf7a expression across the various tissues in fly, however, the underlying pathomechanisms are likely different. Based on available literature for SARS-CoV-2 and related SARS-CoV proteins, we can make some predictions as to the pathways affected by each. Orf6 has been known to have interferon (IFN) antagonistic properties based on previous coronaviruses. This function has been retained by SARS-CoV-2 Orf6 [23, 24], with ability to interfere with interferon production comparable to its SARS-CoV equivalent [41]. SARS-CoV Orf6 has been shown to inhibit primary interferon production [18] and to antagonize STAT1 function (interferon signaling) by altering the host nuclear import factors [9]. Indeed, interaction of SARS-CoV-2 Orf6 with proteins of the nuclear pore complex [10] and its effect on IFN stimulated genes (ISG) [28] have been reported recently. The interaction with the nuclear pore complex is evident from our data as well (Selinexor; Fig. 6). SARS-CoV Nsp6 localizes to the endoplasmic reticulum, where it has been shown to interact with Nsp3 and Nsp4 to induce double-membrane vesicles [1], and its SARS-CoV-2 equivalent has been predicted to interact with multiple ATPases of vesicle trafficking [10]. It might also interact with SIGMA1R, a receptor thought to regulate the endoplasmic reticulum stress response [10]. SARS-CoV-2 Orf7a protein has been predicted to interact with ribosomal transport proteins HEATR3 and MDN1 [10]. Previously, SARS-CoV Orf7a has been demonstrated to inhibit cellular translation and induce apoptosis [19], in line with our viability data for SARS-CoV-2 Orf7a. The SARS-CoV protein was shown to interact with Bcl-X_L_ and other pro-survival proteins (Bcl-2, Bcl-2, Mcl-1, and A1), but not with pro-apoptotic proteins (Bax, Bak, Bad, and Bid) [35]. The findings suggested Orf7a might trigger apoptosis by interfering directly with the pro-survival function of Bcl-X_L_, indeed both proteins were shown to co-localize at the endoplasmic reticulum and mitochondria [35]. Together, these findings indicate that even though the SARS-CoV-2 Orf6, Nsp6 and Orf7a proteins lead to similar viability defects and tissue damage, the underlying pathomechanisms are likely different.

SARS-CoV-2 Orf6 has been shown to act as an antagonist of IFN signaling [23, 24, 27, 41] as well as to directly interact with the NUP98-RAE1 complex at the nuclear pore [10, 23, 24, 27]. We similarly report SARS-CoV-2 Orf6 protein–protein interaction with many key members of the nuclear pore machinery, including nucleoporins and karyopherins (both importins and exportins) [22]. Selinexor, an FDA-approved selective inhibitor of nuclear export [33], has been predicted to disrupt this interaction of SARS-CoV-2 Orf6 with the host nuclear pore complex [10]. We report it reduced SARS-CoV-2 Orf6 cytotoxic effects in a human in vitro culture model [22]. Notably, the nuclear pore proteins in the Orf6 interaction network are extremely conserved between human and fly (Fig. 1) [22], therefore we similarly treated the flies with this nuclear export inhibitor. Indeed, Selinexor treatment greatly attenuated the Orf6-induced muscle and trachea (lung) defects observed in fly (Figs. 5 and 6). However, even though we found phenotypic overlap between Orf6, Orf7a and Nsp6 in our assays, Selinexor was unable to counteract Orf7a and Nsp6 effects, supporting the notion that the viral proteins act through different pathomechanisms. These findings further underpin our hypothesis that inhibition of viral entry/replication (the strategy of COVID-19 interventions like Remdesivir) might proof insufficient by itself as the approach might stop the virus in its tracks but does not remedy the multi-tissue effects of virus already present in the host/pateint. Therefore, we propose to use our Orf6 findings as a starting point to identify additional virus-host pathogenic interactions, then to develop drugs to target and disrupt these interactions. Therapeutic strategies, combining inhibition of virus entry/replication and pathogenic host interactions, would both stop SARS-CoV-2 spread and ameliorate the devastating progression of COVID-19 symptomatology.

In the current study, we have identified SARS-CoV-2 primary determinant pathogenic proteins (Orf6, Nsp6 and Orf7a). Capitalizing on the whole fly, enabled us to study the pathogenic effects of the individual viral proteins on muscle and trachea (lung). We expanded our understanding of the pathomechanism invoked by Orf6 and showed that Selinexor was able to attenuate Orf6 pathogenicity specifically in vivo. Together with the high conservation across the SARS-CoV-2 virus-host protein interaction network between human and fly, these data strongly support the fly as an effective model system for studying individual SARS-CoV-2 proteins.

## Methods

### Conservation SARS-CoV-2 and human host interactome in *Drosophila*

We used the DRSC Integrative Ortholog Prediction Tool (DIOPT) version 8 [13] for the identification of fly orthologs for the SARS-CoV-2 binding human proteins. DIOPT summarizes heterogeneous sources of conservation study tools and databases, providing integrative ortholog predictions, where score 0 indicates no orthologs and score 16 indicates all sources predicted the human-fly ortholog pair. Cytoscape version 3.8.0 [32] was used to map the fly ortholog information (i.e. DIOPT scores) on to the SARS-CoV-2 human host interactome [10].

### Fly strains

All fly stocks were reared and kept on standard food at 25 °C. The fly lines were obtained from the Bloomington Drosophila Stock Center (Indiana University, IN): *w*^1118^ (# BL-3605), *UAS*-*SARS-CoV-2 gene* overexpression (OE) strains, *Tubulin-Gal4*/*TM3, Sb* (# BL-5138).

### DNA cloning and generation of transgenic fly strains

The cDNA fragments were PCR-amplified from human codon-optimized SARS-CoV-2 genes (*Nsp1*, *Nsp2*, *Nsp3*, *Nsp6*, *Orf3a*, *Orf3b*, *Orf6*, *Orf7a*, *Orf7b*, *Orf8*, *Orf9b*, and *Orf10*) in pDONR207 vector (from Fritz Roth, through Addgene) or pLVX-EF1alpha-IRES-Puro vector (from Nevan Krogan, through Addgene), and assembled into the pUASTattB vector. The transgenes were introduced into a fixed chromosomal docking site by germ line transformation to generate *UAS*-*SARS-CoV-2-gene* overexpression (OE) transgenic flies.

### Mortality at eclosion

In order to assay the effect of viral genes on viability, a balancer system was used. *UAS*-*SARS-CoV-2 gene* OE flies were crossed with a *Tub-Gal4*/*TM3, Sb* line. Offspring either carry *UAS-SARS-CoV-2 gene OE*/*TM3, Sb* which carry the balancer chromosome resulting in orange eye color, shortened (stubbly; Sb) hairs on the back and no transgene expression, or they carry *UAS-SARS-CoV-2 gene OE*/*Tub-Gal4*, resulting in expression of the SARS-CoV-2 gene driven by *Tub-Gal4* and transgenic flies with the typical red eyes and long hair (Fig. 2a). Embryo progeny were collected and allowed to develop under standard conditions. Mortality at eclosion (adult emergence from pupa stage) was based on the percentage of flies with SARS-CoV-2 gene expression (red, long) that failed to emerge as adults, relative to siblings that did not express the SARS-CoV-2 gene construct (orange, short). The result was presented as a Mortality Index calculated as: (long hair − short hair) / short hair × 100.

### Adult survival assay

Following egg laying, *Drosophila* larvae were kept at 25 °C, standard conditions and an optimal temperature for *UAS*-transgene expression. Adult male flies were maintained in vials in groups of 20 or fewer. Number of life flies in each group was recorded every second day. *Drosophila* lifespan is typically 50–60 days for wild type flies. The assay was ended when no survivors were left for any of the transgenic lines. A 100 flies were assayed per genotype.

### Climbing assay

Climbing male flies were monitored by analyzing their performance to climb 6 cm in a horizontal tube within 14 s. A successful attempt was scored as 1, and failure to reach the top (6 cm line) as 0. Each fly was assessed five times to calculate the average climbing score. At least 30 flies per genotype were analyzed.

### *Drosophila* wing observation

Wing position was observed using a ZEISS Stemi 305 Stereo Zoom microscope with 5:1 zoom. A total of 200 flies were counted (4 experimental replicates, 50 flies per group each time). Representative images were taken using a ZEISS SteREO Discovery.V12, modular stereo microscope with 12 × zoom.

### *Drosophila* indirect flight muscle imaging

Flies were dissected and fixed for 30 min in 4% paraformaldehyde in phosphate-buffered saline (1 × PBS). Indirect flight muscle fibers from both wings were assayed. Alexa Fluor 647 phalloidin was obtained from Thermo Fisher. To label mitochondria, mouse anti-Atp5a antibody was used at 1:500, followed by Alexa Fluor 488-conjugated secondary antibodies. Confocal imaging of the *Drosophila* indirect fly muscle was performed using a ZEISS LSM900 confocal microscope with a 63 × Plan-Apochromat 0.8 N.A. oil objective. For quantitative comparisons of fluorescence intensity, common settings were chosen to avoid oversaturation. ImageJ Software (version 1.52a) [31] was used to process images. Mitochondria were counted manually from the images, based on visual observation of rounded morphology separating individual segments (see Fig. 6c, *w*^1118^ for example).

### Imaging *Drosophila* tracheal branching

For tracheal observations, 3rd instar larvae were dissected as described previously [5]. Six larvae were dissected for each group. Images of the tracheal branches for each were obtained using a using a ZEISS SteREO Discovery.V12, modular stereo microscope with 12 × zoom. Tracheal branches in each segment were counted manually.

### Selinexor treatment

Selinexor (Selleck, # KPT-330) was dissolved in dimethyl sulfoxide (DMSO; Sigma) and added to standard fly food at indicated doses (0.1, 0.2, and 0.5 μM). For untreated, control (0 μM), DMSO alone was added to the food. Flies were treated from first instar larval stage until the adult flies hatched.

### Statistical analysis

Statistical tests were performed using PAST.exe software (http://folk.uio.no/ohammer/past/index.html) unless otherwise noted. Data were first tested for normality by using the Shapiro–Wilk test (a = 0.05). Normally distributed data were analyzed either by Student’s t-test (two groups) and Bonferroni comparison to adjust the P value, or by a one-way analysis of variance followed by a Tukey–Kramer post-test for comparing multiple groups. Non-normal distributed data were analyzed by either a Mann–Whitney test (two groups) and Bonferroni comparison to adjust P value, or a Kruskal–Wallis H-test followed by a Dunn’s test for comparisons between multiple groups. Statistical significance was defined as P < 0.05.

## Supplementary Information

**Additional file 1. Table S1.** Conservation scores for SARS-CoV-2 human protein-protein host interactome in Drosophila.

## Data Availability

All data and materials generated in this study are available publicly upon request.
