# Supplementary material for: Functional analysis of SARS-CoV-2 proteins in Drosophila identifies Orf6-induced pathogenic effects with Selinexor as an effective treatment
Source: Cell Biosci. 2021 Mar 25;11:59. doi: 10.1186/s13578-021-00567-8 (PMC7992514; doi:10.1186/s13578-021-00567-8)
Supplement: Supplementary file 1 — Additional file 1. Table S1. Conservation scores for SARS-CoV-2 human protein-protein host interactome in Drosophila. [file 13578_2021_567_MOESM1_ESM.pdf]

Supplemental Table S1. Conservation scores for SARS-CoV-2 human protein-protein host interactome in *Drosophila*.

Viral Protein	Human Protein	DIOPT Score	Fly Ortholog	Fly Flybase ID
Nsp1	POLA1	15	DNApol-alpha180	FBgn0259113
Nsp1	PRIM1	14	DNApol-alpha50	FBgn0011762
Nsp1	PRIM2	14	DNApol-alpha60	FBgn0259676
Nsp1	POLA2	15	DNApol-alpha73	FBgn0005696
Nsp1	COLGALT1	12	CG31915	FBgn0051915
Nsp1	PKP2	4	p120ctn	FBgn0260799
Nsp2	SLC27A2	6	Fatp2	FBgn0265187
Nsp2	EIF4E2	13	eIF4EHP	FBgn0053100
Nsp2	POR	14	Cpr	FBgn0015623
Nsp2	RAP1GDS1	15	vimar	FBgn0022960
Nsp2	WASHC4	14	SWIP	FBgn0030739
Nsp2	FKBP15	4	CG3342	FBgn0029874
Nsp2	GIGYF2	10	Gyf	FBgn0039936
Nsp4	IDE	14	Ide	FBgn0001247
Nsp4	TIMM10	14	Tim10	FBgn0027360
Nsp4	ALG11	13	Alg11	FBgn0037108
Nsp4	NUP210	15	Gp210	FBgn0266580
Nsp4	TIMM29	13	CG14270	FBgn0029665
Nsp4	DNAJC11	14	CG8531	FBgn0033918
Nsp4	TIMM10B	11	Tim9b	FBgn0027358
Nsp4	TIMM9	15	Tim9a	FBgn0030480
Nsp5	HDAC2	13	HDAC1	FBgn0015805
Nsp5_C145A	GPX1	3	CG15116	FBgn0034415
Nsp5_C145A	TRMT1	15	CG6388	FBgn0032430
Nsp6	ATP5MG	15	ATPsynG	FBgn0010612
Nsp6	ATP6AP1	9	VhaAC45	FBgn0262515
Nsp6	SIGMAR1	1	CG43446	FBgn0263400
Nsp6	ATP13A3	13	anne	FBgn0052000
Nsp7	AGPS	14	ADPS	FBgn0033983
Nsp7	CYB5B	13	Cyt-b5	FBgn0264294
Nsp7	ACSL3	14	Acsl	FBgn0263120
Nsp7	CYB5R3	11	CG5946	FBgn0036211
Nsp7	RALA	13	Rala	FBgn0015286
Nsp7	COMT	0		
Nsp7	RAB5C	13	Rab5	FBgn0014010
Nsp7	RAB7A	13	Rab7	FBgn0015795
Nsp7	RAB8A	13	Rab8	FBgn0262518
Nsp7	RAB2A	13	Rab2	FBgn0014009
Nsp7	RAB10	12	Rab10	FBgn0015789
Nsp7	RAB14	13	Rab14	FBgn0015791
Nsp7	RHOA	13	Rho1	FBgn0014020
Nsp7	RAB1A	13	Rab1	FBgn0285937
Nsp7	GNB1	15	Gbeta13F	FBgn0001105
Nsp7	GNG5	8	Ggamma1	FBgn0004921

Nsp7	LMAN2	13	CG5510	FBgn0039160
Nsp7	MOGS	15	GCS1	FBgn0030289
Nsp7	TOR1AIP1	1	TORIP	FBgn0036908
Nsp7	MTARC1	13	Marc	FBgn0033451
Nsp7	QSOX2	15	Qsox2	FBgn0038919
Nsp7	HS2ST1	14	Hs2st	FBgn0024230
Nsp7	NDUFAF2	6	CG43346	FBgn0263051
Nsp7	SCCPDH	15	Sccpdh1::Sccpdh	FBgn0037298::FBgn0038038
Nsp7	SCARB1	8	crq::emp	FBgn0010435::FBgn0015924
Nsp7	NAT14	1	CG18542	FBgn0037731
Nsp7	DCAKD	15	Dpck	FBgn0037469
Nsp7	FAM162A	11	CG9231	FBgn0036887
Nsp7	DNAJC19	13	CG7394	FBgn0036173
Nsp7	SELENOS	0		
Nsp7	PTGES2	14	Su(P)	FBgn0004465
Nsp7	RAB18	13	Rab18	FBgn0015794
Nsp8	MPHOSPH10	12	CG13097	FBgn0032051
Nsp8	SRP72	14	Srp72	FBgn0038810
Nsp8	ATE1	14	Ate1	FBgn0025720
Nsp8	NSD2	12	NSD	FBgn0039559
Nsp8	SRP19	14	Srp19	FBgn0015298
Nsp8	SRP54	11	Srp54k	FBgn0010747
Nsp8	MRPS25	13	mRpS25	FBgn0030572
Nsp8	DDX10	14	CG5800	FBgn0030855
Nsp8	LARP7	10	Larp7	FBgn0260771
Nsp8	MEPCE	11	CG1239	FBgn0037368
Nsp8	NGDN	14	CG11030	FBgn0031736
Nsp8	EXOSC8	2	Rrp42::Rrp45	FBgn0030789::FBgn0034065
Nsp8	NARS2	15	AsnRS-m	FBgn0034177
Nsp8	NOL10	14	l(2)34Fd	FBgn0261535
Nsp8	CCDC86	8	CG14210	FBgn0031040
Nsp8	SEPSECS	13	CG1427	FBgn0037347
Nsp8	EXOSC5	15	Rrp46	FBgn0037815
Nsp8	EXOSC3	15	Rrp40	FBgn0260648
Nsp8	AATF	12	Aatf	FBgn0031851
Nsp8	HECTD1	15	Ufd4	FBgn0032208
Nsp8	MRPS2	14	mRpS2	FBgn0031639
Nsp8	MRPS5	14	mRpS5	FBgn0044510
Nsp8	EXOSC2	15	Rrp4	FBgn0034879
Nsp8	MRPS27	9	CG4882	FBgn0025336
Nsp9	GTF2F2	15	TfIIFbeta	FBgn0010421
Nsp9	FBN1	3	dpy::frac	FBgn0035798::FBgn0053196
Nsp9	FBN2	4	dpy	FBgn0053196
Nsp9	NUP214	10	Nup214	FBgn0010660
Nsp9	NUP62	10	Nup62	FBgn0034118
Nsp9	DCAF7	13	wap	FBgn0266848
Nsp9	EIF4H	13	eIF4H1	FBgn0262734
Nsp9	NUP54	12	Nup54	FBgn0033737

Nsp9	MIB1	15	mib1	FBgn0263601
Nsp9	SPART	13	spartin	FBgn0037265
Nsp9	NEK9	9	niki	FBgn0045980
Nsp9	ZNF503	13	noc	FBgn0005771
Nsp9	NUP88	14	mbo	FBgn0026207
Nsp9	NUP58	12	Nup58	FBgn0038722
Nsp9	MAT2B	3	CG5854	FBgn0039130
Nsp9	FBLN5	3	frac	FBgn0035798
Nsp10	AP2A2	13	AP-2alpha	FBgn0264855
Nsp10	GFER	12	Alr	FBgn0031068
Nsp10	ERGIC1	2	CG4293::CG7011	FBgn0024983::FBgn0036489
Nsp10	AP2M1	13	AP-2mu	FBgn0263351
Nsp10	GRPEL1	14	Roe1	FBgn0014877
Nsp11	TBCA	15	CG1890	FBgn0039869
Nsp12	SBNO1	13	sno	FBgn0265630
Nsp12	BCKDK	2	Pdk	FBgn0017558
Nsp12	AKAP8	0		
Nsp12	MYCBP2	14	hiw	FBgn0030600
Nsp12	SLU7	14	Slu7	FBgn0039626
Nsp12	RIPK1	2	slpr::Tak1	FBgn0026323::FBgn0030018
Nsp12	UBAP2L	10	lig	FBgn0020279
Nsp12	TYSND1	13	CG3589	FBgn0035065
Nsp12	PDZD11	2	veli	FBgn0039269
Nsp12	PRRC2B	4	nocte	FBgn0261710
Nsp12	UBAP2	11	lig	FBgn0020279
Nsp12	ZNF318	8	CG9776	FBgn0027866
Nsp12	CRTC3	7	Crtc	FBgn0036746
Nsp12	USP54	6	ec	FBgn0000542
Nsp12	ZC3H7A	0		
Nsp12	LARP4B	7	Larp4B	FBgn0035424
Nsp12	RBM41	2	CG44249::Snp	FBgn0265184::FBgn0265192
Nsp12	TCF12	12	da	FBgn0267821
Nsp12	PPIL3	14	CG11777	FBgn0033527
Nsp12	PLEKHA5	5	CG34383	FBgn0085412
Nsp13	TBKBP1	0		
Nsp13	CIT	12	sti	FBgn0002466
Nsp13	HSBP1	12	CG5446	FBgn0032429
Nsp13	PCNT	6	Plp	FBgn0086690
Nsp13	CEP43	0		
Nsp13	PRKAR2A	13	Pka-R2	FBgn0022382
Nsp13	PRKACA	13	Pka-C1	FBgn0000273
Nsp13	PRKAR2B	11	Pka-R2	FBgn0022382
Nsp13	RDX	13	Moe	FBgn0011661
Nsp13	CENPF	1	cana::cmet::Golgin245::lva::mud	FBgn0002873::FBgn0029688::FBgn0034854::FBgn0040232::FBgn0040233
Nsp13	TLE1	11	gro	FBgn0001139
Nsp13	TLE3	12	gro	FBgn0001139
Nsp13	TLE5	4	gro	FBgn0001139

Nsp13	GOLGA3	2	GCC185	FBgn0037979
Nsp13	GOLGA2	9	GM130	FBgn0034697
Nsp13	GOLGB1	2	Golgin245	FBgn0034854
Nsp13	GRIPAP1	0		
Nsp13	CEP350	1	CG9279::CLIP-190::DCTN1-p150	FBgn0001108::FBgn0020503::FBgn0036882
Nsp13	PDE4DIP	2	cnn	FBgn0013765
Nsp13	CEP135	12	Cep135	FBgn0036480
Nsp13	CEP68	0		
Nsp13	CNTRL	1	Cep97::CG10839::CG13708::CG14185::CG8800::Ppr-Y::sds22::TbCMF46::tilB	FBgn0014395::FBgn0028858::FBgn0028992::FBgn0031575::FBgn0032163::FBgn0033408::FBgn0035577::FBgn0036936::FBgn0046697
Nsp13	ERC1	6	brp	FBgn0259246
Nsp13	GCC2	10	GCC185	FBgn0037979
Nsp13	CLIP4	2	CG9279::CLIP-	FBgn0020503::FBgn0036882
Nsp13	NIN	7	Bsg25D	FBgn0000228
Nsp13	CEP112	0		
Nsp13	MIPOL1	0		
Nsp13	USP13	12	Usp5	FBgn0035402
Nsp13	GCC1	14	GCC88	FBgn0037881
Nsp13	JAKMIP1	2	Pif1A	FBgn0261015
Nsp13	CDK5RAP2	5	cnn	FBgn0013765
Nsp13	AKAP9	5	Plp	FBgn0086690
Nsp13	GORASP1	8	Grasp65	FBgn0036919
Nsp13	FYCO1	2	rush	FBgn0025381
Nsp13	C1orf50	11	CG31800	FBgn0051800
Nsp13	CEP250	7	Root	FBgn0039152
Nsp13	TBK1	13	IKKepsilon	FBgn0086657
Nsp13	HOOK1	10	hook	FBgn0001202
Nsp13	NINL	8	Bsg25D	FBgn0000228
Nsp14	GLA	12	CG7997	FBgn0034117
Nsp14	IMPDH2	10	ras	FBgn0003204
Nsp14	SIRT5	1	Sirt1::Sirt2::Sirt4::Sirt6::Sirt7	FBgn0024291::FBgn0029783::FBgn0037802::FBgn0038788::FBgn0039631
Nsp15	NUTF2	15	Ntf-2	FBgn0031145
Nsp15	ARF6	15	Arf51F	FBgn0013750
Nsp15	RNF41	15	elgi	FBgn0283649
Spike	GOLGA7	12	CG5447	FBgn0039427
Spike	ZDHHC5	10	CG34449	FBgn0085478
E	AP3B1	13	rb	FBgn0003210
E	BRD4	9	fs(1)h	FBgn0004656
E	BRD2	9	fs(1)h	FBgn0004656
E	CWC27	13	CG10907	FBgn0036207
E	ZC3H18	11	CG1677	FBgn0029941
E	SLC44A2	13	Ctl2	FBgn0039637
M	PMPCB	15	UQCR-C1	FBgn0038271

M	YIF1A	12	Yif1	FBgn0039450
M	ATP1B1	14	nrv1::nrv3	FBgn0015776::FBgn0032946
M	ACADM	14	Mcad	FBgn0035811
M	ETFA	12	wal	FBgn0010516
M	STOM	12	CG42540	FBgn0260657
M	GGCX	14	GC	FBgn0035245
M	ATP6V1A	13	Vha68-1::Vha68-2	FBgn0263598::FBgn0265262
M	PSMD8	15	Rpn12	FBgn0028693
M	REEP5	14	ReepB	FBgn0033906
M	PMPCA	15	CG8728	FBgn0033235
M	ANO6	8	CG6938	FBgn0036235
M	PITRM1	15	CG3107	FBgn0033005
M	SLC30A9	15	ZnT49B	FBgn0033762
M	FASTKD5	9	CG2124	FBgn0030217
M	SLC30A7	15	ZnT86D	FBgn0037875
M	TUBGCP3	15	Grip91	FBgn0001612
M	COQ8B	15	Coq8	FBgn0052649
M	SAAL1	7	CG30467	FBgn0050467
M	REEP6	11	ReepB	FBgn0033906
M	INTS4	14	IntS4	FBgn0026679
M	SLC25A21	15	CG5254	FBgn0040383
M	TUBGCP2	15	Grip84	FBgn0026430
M	TARS2	6	ThrRS	FBgn0027081
M	RTN4	9	Rtnl1	FBgn0053113
M	FAM8A1	12	CG8237	FBgn0033350
M	AASS	15	LKRSDH	FBgn0286198
M	AKAP8L	0		
M	AAR2	11	CG12320	FBgn0038590
M	BZW2	12	kra	FBgn0250753
N	RRP9	14	U3-55K	FBgn0053505
N	PABPC1	12	pAbp	FBgn0265297
N	CSNK2A2	7	CkIIalpha	FBgn0264492
N	CSNK2B	13	CkIIbeta	FBgn0000259
N	G3BP1	12	rin	FBgn0015778
N	PABPC4	13	pAbp	FBgn0265297
N	LARP1	10	larp	FBgn0261618
N	FAM98A	12	CG5913	FBgn0039385
N	SNIP1	12	CG17168	FBgn0039943
N	UPF1	14	Upf1	FBgn0030354
N	MOV10	8	CG6967	FBgn0034187
N	G3BP2	10	rin	FBgn0015778
N	DDX21	1	CG10077::CG14443::mahe::Rm62	FBgn0003261::FBgn0029880::FBgn0029979::FBgn003572
N	RBM28	12	CG4806	FBgn0260456
N	RPL36	10	RpL36	FBgn0002579
Orf3a	HMOX1	10	Ho	FBgn0037933
Orf3a	TRIM59	3	Oseg5	FBgn0032891
Orf3a	ARL6IP6	5	CG8321	FBgn0033677

Orf3a	VPS39	14	Vps39	FBgn0038593
Orf3a	CLCC1	0		
Orf3a	VPS11	13	Vps11	FBgn0052350
Orf3a	SUN2	6	koi	FBgn0265003
Orf3a	ALG5	14	wol	FBgn0261020
Orf3b	STOML2	13	Stoml2	FBgn0034936
Orf6	NUP98	12	Nup98-96	FBgn0039120
Orf6	RAE1	13	Rae1	FBgn0034646
Orf6	MTCH1	12	Mtch	FBgn0027786
Orf7a	HEATR3	14	CG10286	FBgn0037439
Orf7a	MDN1	13	CG13185	FBgn0033661
Orf8	PLOD2	12	Plod	FBgn0036147
Orf8	TOR1A	15	Torsin	FBgn0025615
Orf8	STC2	0		
Orf8	PLAT	1	CG6462::CG6592::CG7432::flz::Nrk	FBgn0020391::FBgn0035663::FBgn0035669::FBgn0038727::FBgn0286782
Orf8	ITGB1	14	mys	FBgn0004657
Orf8	CISD3	12	CG3420	FBgn0033100
Orf8	COL6A1	1	Mp	FBgn0260660
Orf8	PVR	2	Fas3	FBgn0000636
Orf8	DNMT1	1	Mt2	FBgn0028707
Orf8	LOX	3	Loxl1::Loxl2	FBgn0034660::FBgn0039848
Orf8	PCSK6	7	Fur2	FBgn0004598
Orf8	INHBE	8	Actbeta	FBgn0024913
Orf8	NPC2	13	Npc2a	FBgn0031381
Orf8	MFGE8	0		
Orf8	OS9	3	CG6766	FBgn0032398
Orf8	NPTX1	2	b6	FBgn0024897
Orf8	POGLUT2	3	CG31139	FBgn0051139
Orf8	POGLUT3	2	CG31139	FBgn0051139
Orf8	ERO1B	13	Ero1L	FBgn0261274
Orf8	PLD3	15	CG9248	FBgn0032923
Orf8	FOXRED2	1	Fmo-1::Fmo-2	FBgn0033079::FBgn0034943
Orf8	CHPF	14	CG43313	FBgn0263005
Orf8	PUSL1	13	CG34140	FBgn0083976
Orf8	EMC1	13	EMC1	FBgn0037530
Orf8	GGH	15	l(3)72Dp	FBgn0263607
Orf8	ERLEC1	14	CG6766	FBgn0032398
Orf8	IL17RA	0		
Orf8	NGLY1	15	Pngl	FBgn0033050
Orf8	HS6ST2	11	Hs6st	FBgn0038755
Orf8	SDF2	14	CG11999	FBgn0037312
Orf8	NEU1	0		
Orf8	GDF15	3	mav	FBgn0039914
Orf8	TM2D3	12	amx	FBgn0000077
Orf8	ERP44	15	CG9911	FBgn0030734
Orf8	EDEM3	13	Edem2	FBgn0032480
Orf8	SIL1	13	Sil1	FBgn0039296

Orf8	POFUT1	13	O-fut1	FBgn0033901
Orf8	SMOC1	13	magu	FBgn0262169
Orf8	PLEKHF2	15	rush	FBgn0025381
Orf8	FBXL12	2	CG12402::CG148<br>91::CG5003::CG9	FBgn0032878::FBgn0038202<br>::FBgn0038445::FBgn003955
Orf8	UGGT2	13	Uggt	FBgn0014075
Orf8	CHPF2	12	CG43313	FBgn0263005
Orf8	ADAMTS1	7	AdamTS-A	FBgn0286071
Orf8	HYOU1	15	CG2918	FBgn0023529
Orf8	FKBP7	4	Fkbp14	FBgn0010470
Orf8	ADAM9	7	Meltrin	FBgn0265140
Orf8	FKBP10	2	CG14715::Fkbp14<br>::Fkbp39	FBgn0010470::FBgn0013269<br>::FBgn0037930
Orf9b	SLC9A3R1	9	CG10939	FBgn0010620
Orf9b	CHMP2A	13	Vps2	FBgn0039402
Orf9b	CSDE1	15	Unr	FBgn0263352
Orf9b	TOMM70	15	Tom70	FBgn0032397
Orf9b	MARK3	12	par-1	FBgn0260934
Orf9b	MARK2	9	par-1	FBgn0260934
Orf9b	DPH5	13	Dph5	FBgn0024558
Orf9b	DCTPP1	0		
Orf9b	MARK1	10	par-1	FBgn0260934
Orf9b	PTBP2	13	heph	FBgn0011224
Orf9b	BAG5	3	stv	FBgn0086708
Orf10	PPT1	14	Ppt1	FBgn0030057
Orf10	CUL2	14	Cul2	FBgn0032956
Orf10	MAP7D1	4	ens	FBgn0264693
Orf10	THTPA	0		
Orf10	ZYG11B	2	CG12084	FBgn0043458
Orf10	TIMM8B	14	Tim8	FBgn0027359
Orf10	RBX1	15	Roc1a	FBgn0025638
Orf10	ELOC	15	EloC	FBgn0266711
Orf10	ELOB	11	EloB	FBgn0023212
